# The effect of patient satisfaction scores on physician job satisfaction and burnout

**DOI:** 10.2144/fsoa-2020-0136

**Published:** 2020-11-12

**Authors:** Byron J Schneider, Reza Ehsanian, Alex Schmidt, Lisa Huynh, David J Kennedy, Dermot P Maher, Sterling Haring

**Affiliations:** 1 Department of Physical Medicine & Rehabilitation, Center for Musculoskeletal Research, Vanderbilt University Medical Center, Nashville, TN 37212, USA; 2 Department of Neurosurgery, Division of Physical Medicine & Rehabilitation, University of New Mexico School of Medicine, Albuquerque, NM 87106, USA; 3 Department of Physical Medicine & Rehabilitation, Vanderbilt University Medical Center, Nashville, TN 37212, USA; 4 Department of Orthopaedic Surgery, Stanford University, Stanford, CA 94063, USA; 5 Department of Anesthesiology & Critical Care Medicine, Johns Hopkins University, Baltimore, MD 21287, USA; 6 Department of Health Policy & Management, Johns Hopkins Bloomberg School of Public Health, Johns Hopkins University, Baltimore, MD 21205, USA

**Keywords:** burnout, job satisfaction, pain, patient satisfaction, physicians, professional

## Abstract

Physician burnout is recognized as reversible with the potential to negatively influence quality of care and patient outcomes. The study objective was to evaluate associations between patient satisfaction scores (PSS) and physicians’ perceptions of job satisfaction and burnout via a physician survey. Eighty two out of 107 report PSS are institutionally tracked, with 23/107 and 39/107 reporting PSS utilization in financial compensation or performance review, respectively. Fifty four out of 107, report pressure to emphasize PSS; 63/107, report PSS having negative effect on job satisfaction; 31/107 considered leaving their job or career due to PSS and 84/107 report PSS contribute to burnout. In the cohort of physicians treating patients with spine pain who responded to this survey, PSS are associated with decreased job satisfaction and increased burnout.

## Lay abstract

Physician burnout can have a negative impact on the quality of care provided to patients as well as medical outcomes of patients. The current study investigates if patient satisfaction surveys are associated with physician perceptions of job satisfaction and burnout. The study reveals that there may be an association between patient satisfaction surveys and decreases in job satisfaction as well as increases in burnout of physicians. Although no definitive correlation (i.e., we cannot prove that one thing causes another) can be made from this study, it underscores the need for further research and careful analysis of the impact of these metrics (patient satisfaction surveys) on the well-being of physicians.

Physician burnout is increasingly recognized as a reversible state with the potential to negatively influence quality of care and patient outcomes [1]. The prevalence of physician burnout has been reported to be as high as 50% [2]. The inter-relationship between depression, burnout and physician suicide has garnered increased attention. Factors extrinsic to the physician may also play key roles in the development of burnout [3,4].

Particularly since the passage of the Patient Protection and Affordable Care Act 2010, patient satisfaction metrics have been used as a measure of healthcare quality, with mixed results [5,6]. Medicare now requires patient satisfaction be collected formally via the Consumer Assessment of Healthcare Providers and Systems [3,4]. Other standardized reporting proprietary questionnaires may be solicited from the patient by an institution, such as that distributed by Press Ganey^®^, to a random sample of treated patients [7]. As insurers move toward alternative payment models, these scores have been cited in performance evaluations and metrics related to physician compensation [8,9].

Since the Institute of Medicine released its landmark publication, Crossing the Quality Chasm in 2001, patient-centered care has become a central tenet of 21st century medicine [10]. While undoubtedly a worthy goal, the establishment of a working definition and, therefore, measurement of success and failure, have proven elusive [11–13]. In the absence of validated, outcome-anchored instruments directly measuring patient-centered care, patient satisfaction has become a proxy measure of choice. Accordingly, patient satisfaction surveys are routinely distributed throughout health systems and have been tied to incentives, including accreditation and reimbursement [14–16]. While many of the ethical challenges posed by patient-centeredness have been explored in the literature, the real-world, practice-altering implications are less clear [16,17]. Perhaps nowhere are these conflicts more apparent than in the practice of pain medicine, wherein the quest for patient satisfaction may be complicated by treatments offered, such as prescribing opioid medications [18–20]. The associations between patient satisfaction scores (PSS) and health outcomes, healthcare received and physician satisfaction have been previously reported [18,19,21–23]. There is a paucity of published data on the effects of PSS on physician well-being. Of particular interest, it is unknown whether this is a contributor to physician burnout.

Given the current focus on physician burnout and the ubiquity of patient satisfaction surveys, we sought to quantify and characterize the relationship between the utilization of PSS and perceptions of job satisfaction, burnout and career planning among physicians treating patients with spine pain. The main purpose of the study was to evaluate associations between PSS and physicians’ perceptions of job satisfaction and burnout factors.

## Materials & methods

A 23-question survey was developed and agreed upon by physicians at multiple institutions, with the primary goal of assessing physicians’ beliefs and behaviors pertinent to PSS. In addition to collecting demographics, questions were focused on two primary issues: healthcare utilization (eight questions) and physician job satisfaction (six questions). The full version of the survey is available (Supplementary Figure 1). In this study, we present the results pertinent to physician job satisfaction, the results of healthcare utilization are presented separately. The survey was approved by the Institutional Review Board at the University of the Principal Investigator (BS) and deemed exempt. The survey was then submitted to the Spine Intervention Society (SIS) and was approved for dissemination to its members.

The survey primarily sampled the SIS membership as they best presented the physician population impacted by patient satisfaction surveys. Per the SIS’s informational website “The Spine Intervention Society (SIS) is an international physician organization dedicated to the development and promotion of the highest standards for the practice of interventional pain procedures. Nearly 3000 physician members in 43 countries rely on SIS for its renowned advocacy, research, and educational programs designed to advance evidence-based practice and ensure patient access to quality care and appropriate interventional pain treatments. SIS membership is primarily composed of physicians who are board certified in anesthesiology, physical medicine and rehabilitation, radiology, neurology, orthopedic surgery, or neurosurgery. SIS Mission: To develop and promote the highest standards for the practice of interventional procedures. SIS Vision Interventional procedures that are correctly indicated, competently performed, and beneficial to our patients and society.”

The survey was collected via the REDCAP database at the Principal Investigator’s sponsoring institution. SIS members received unique emails asking for participation in the survey with a respective link. The link was also available to members directly through the SIS website for three consecutive months. The authors of the study also provided a link to the survey and corresponding QR code following three speaking engagements with physician audiences that occurred during the 3-month window.

Statistical analysis consisted of descriptive statistics, which summarized the breakdown of gender, practice setting, primary specialty, location of clinical practice, years of clinical practice as well as parameters affecting the physician related to PSS such as institutional tracking, reimbursement and rating of job performance. In addition descriptive statistics were used to summarize answers to questions from physicians as they relate to PSS such as pressure to consider/focus/emphasize, contribution to physician burnout, and effect on job satisfaction. For each question the number of responses for each selection were divided by the total number of responses for the question.

## Results

The email containing the link to the survey was viewed 1116 times with 223 (20%) clicks on the link. Seventy-five of 223 (34%) of those who clicked on the link completed the survey. An additional 32 physicians accessed the survey directly through the link provided on the SIS website. There was a total of 107 survey participants; with 106/107 (98%) completing all questions and one additional respondent who partially completed the survey, not completing the geographic region of practice.

**Figure 1.**
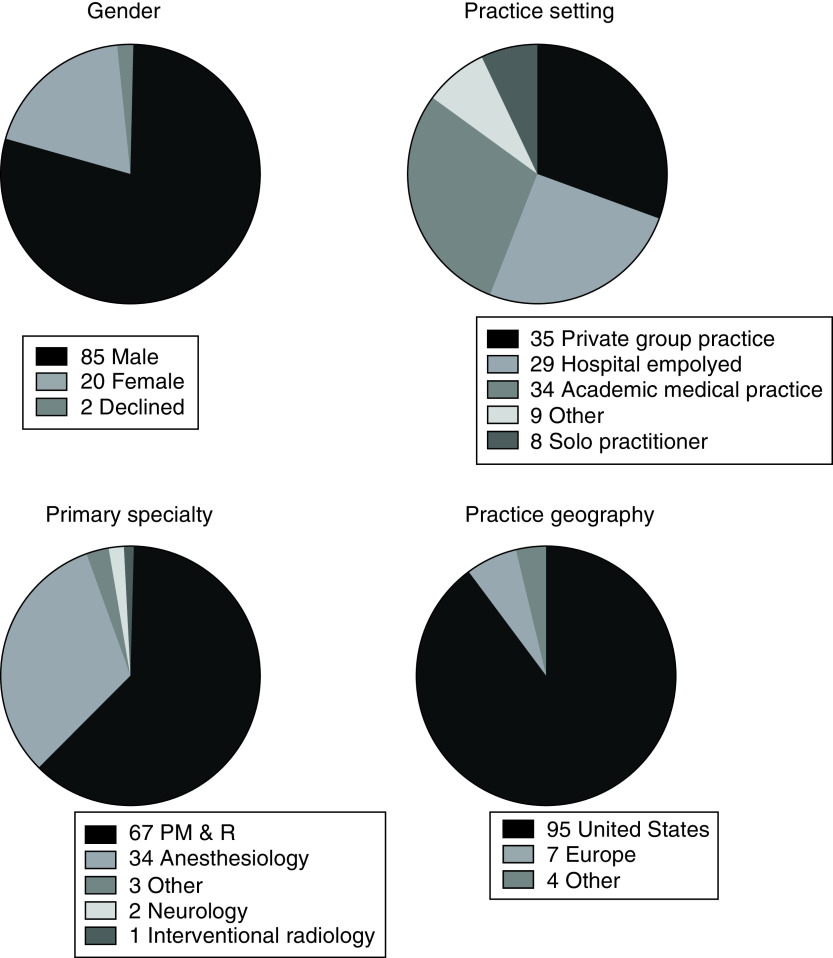
Demographic data of survey respondents (n = 107). PM & R: Physical medicine and rehabilitation.

There were 115 responses, from the 107 participants, to the question enquiring about practice setting. The most common forms of employment were private practice based (35/115; 30%) and hospital based (29/115; 25%); with 30 percent of responds working at an academic medical center (34/115; 30%) (Table 1 & Figure 1). The most common specialties were Physical Medicine and Rehabilitation (67/107; 63%) and Anesthesiology (34/107; 32%). The mean number of years in practice was 14.98 years (standard deviation 10.75), with a median of 12.25 (interquartile range 6–24). The 95% range of responders was 2.25–35 years, with 1 responder reporting 0 which likely represents a trainee. There were 106 responses to the question of geographic location, 95/106 (90%) of responders reporting to be practicing in the US (Table 1 & Figure 1). The majority, 82/107 (77%), of responders reported that their employing institution tracked PSS. Twenty-three of 107 (22%) reported that their reimbursement was tied to PSS and 39/107 (36%) reported that their job performance evaluations were tied to PSS.

**Table 1.** Demographic data of survey respondents (n = 107).

**What is your gender? (107 responses)**	
– Male	85 (79%)
– Female	20 (19%)
– Decline	2 (2%)
**What is your practice setting? (115 responses)**	
– Private group practice	35 (30%)
– Hospital employed	29 (25%)
– Academic medical practice	34 (30%)
– Other	9 (8%)
– Solo practitioner	8 (7%)
– Decline	0 (0%)
**What is your primary specialty? (107 responses)**	
– PM&R	67 (63%)
– Anesthesiology	34 (32%)
– Other	3 (3%)
– Neurology	2 (2%)
– Interventional radiology	1 (1%)
– Internal medicine	0 (0%)
– Surgery	0 (0%)
– Decline	0 (0%)
**Where do you practice? (106 responses)**	
– USA	95 (89.6%)
– Europe	7 (6.6%)
– Other	4 (3.8%)
– Decline	0 (0%)
**How many years have you been in clinical practice?**	
– Average (SD)	14.98 (10.75)
– Median (IQR)	12.25 (6–24)

IQR: Interquartile range; PM&R: Physical medicine and rehabilitation; SD: Standard deviation.

**Figure 2.**
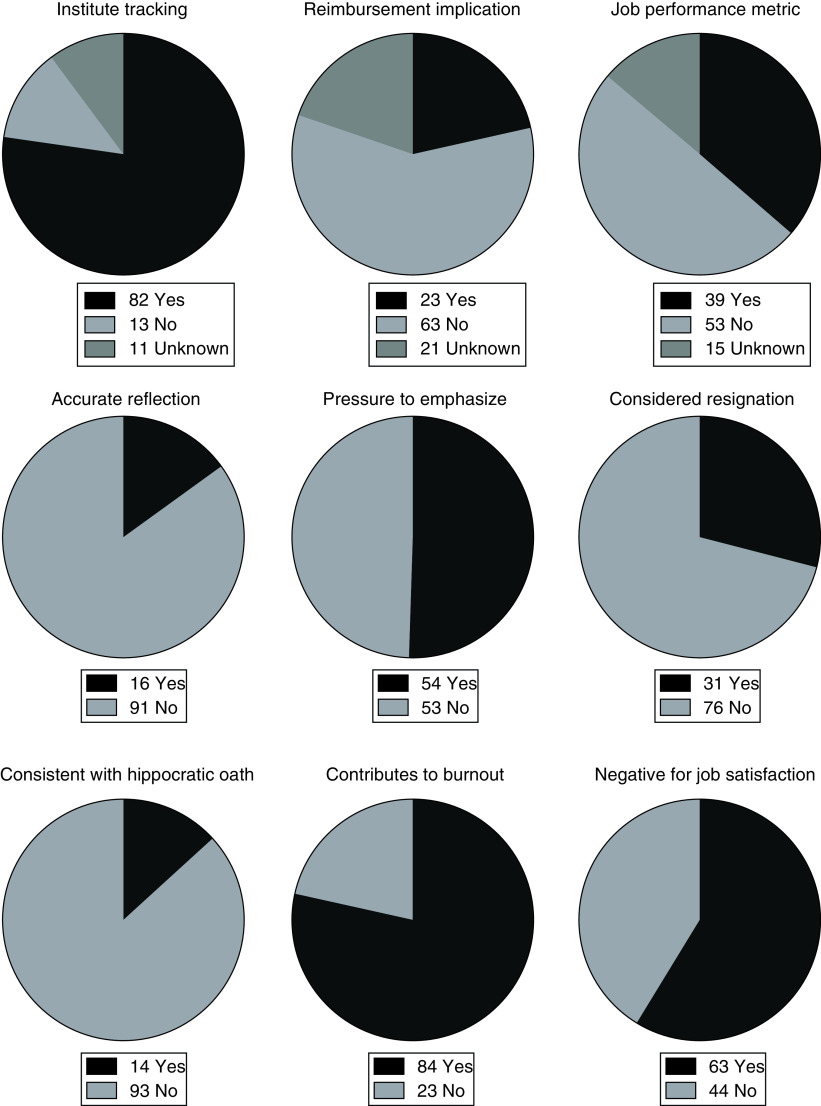
Response related to patient satisfaction scores (n = 107).

Of all respondents, only 16/107 (15%) felt that PSS accurately reflected the quality of care they provided to the patient (Table 2 & Figure 2). Despite this, 54/107 (50%) reported feeling pressure to emphasize PSS (Table 2 & Figure 2). Sixty-three of 107 respondents (59%) reported that PSS have a negative effect on their job satisfaction (Table 2 & Figure 2). Nearly a third (31/107; 29%) reported having considered leaving their job or career because of the current utilization of PSS (Table 2 & Figure 2). Ninety-three of 107 respondents (87%) felt that the emphasis on collection of PSS was inconsistent with the Hippocratic Oath (Table 2 & Figure 2). The majority of respondents (84/107; 79%) felt that the collection and utilization of PSS is contributing to physician burnout (Table 2 & Figure 2).

**Table 2.** Response related to patient satisfaction scores (n = 107).

**Does your institution track patient satisfaction scores?**	
– Yes	82 (77%)
– No	13 (12%)
– Unknown	11 (10%)
**Is your reimbursement tied to your patient satisfaction scores?**	
– Yes	23 (21%)
– No	63 (59%)
– Unknown	21 (20%)
**Are your job performance evaluations tied to patient satisfaction scores?**	
– Yes	39 (36%)
– No	53 (50%)
– Unknown	15 (14%)
**Do you feel that your patient satisfaction scores accurately reflect the quality of your patient care?**	
– Yes	16 (15%)
– No	91 (85%)
– Unknown	0 (0%)
**Do you feel pressured to consider/focus/emphasize patient satisfaction scores?**	
– Yes	54 (50%)
– No	53 (50%)
– Unknown	0 (0%)
**Have you considered leaving your job or career because of how patient satisfaction scores are utilized/collected?**	
– Yes	31 (29%)
– No	76 (71%)
– Unknown	0 (0%)
**Do you believe emphasis on collecting/patient satisfaction scores are consistent with the Hippocratic oath you swore to when becoming a physician?**	
– Yes	14 (13%)
– No	93 (87%)
– Unknown	0 (0%)
**Do you believe the collection of patient satisfaction scores contributes to physician burnout?**	
– Yes	84 (79%)
– No	23 (21%)
– Unknown	0 (0%)
**Does the focus/utilization on patient satisfaction scores negatively affect your job satisfaction?**	
– Yes	63 (59%)
– No	44 (41%)
– Unknown	0 (0%)

## Discussion

Contemporaneous with the adopting of patient-centeredness in healthcare, a rising awareness of physician and trainee burnout has led to increased interest in identifying its prevalence and predictors. Burnout has been associated with elevated rates of job dissatisfaction, early retirement, career change and even physician suicide [24,25].

Despite only 21% of responders in our study reporting that their reimbursement was tied to PSS, nearly three-times as many (59%) reported reduced job satisfaction attributed to collection of PSS. This refutes the argument that there is a financial factor underlying physician discontent regarding patient satisfaction surveys. Furthermore, only 36% of respondents reported that PSS were cited in job performance reviews. This further shows that the collection of PSS alone, and not necessarily how the scores are used, is a potential cause of physician discontent. Indeed, this speaks to a fundamental and unique issue in medicine and particularly in pain medicine. Often patients’ perceptions and desires may be in direct conflict with what is correct and what comprises evidence-based treatment for them.

Perhaps this underscores the alarming finding that a resounding 87% of respondents in this study felt that the emphasis on patient satisfaction was inconsistent with the Hippocratic Oath. PSS have placed physicians in situations in which certain outcomes may be mutually exclusive. As a clinical example, a patient with a normal MRI, who is under the impression they have degenerative disk disease, requesting an epidural steroid injection for symptoms of axial pain. In this and other medical settings, reaffirming the patient’s beliefs and providing the requested treatment is most likely to result in high patient satisfaction, but may also be medically incorrect [18,26–29]. The physician risks reduced patient satisfaction when she or he ‘withholds’ an epidural steroid injection that is not indicated.

It is possible that this discordance is at least one contributor to the finding that the majority of responding physicians (79%) reported increased feelings of burnout associated with PSS. While we did not specifically ask the severity of dissatisfaction and burnout, the fact that nearly a third (29%) reported having considered leaving their job or career due to the current utilization of patient satisfaction data suggest high severity.

Data from other studies support our observations. A Spanish survey of 301 physicians involved in the treatment of pain found that 13% of physicians in pain clinics reported symptoms consistent with burnout compared with a survey average of 7% [30]. Interestingly, burnout among pain physicians was positively associated with pain-related patient outcomes, but negatively associated with outcomes such as patient satisfaction or patient’s quality of life. A separate study of 207 physician members of the American Society of Interventional Pain Physicians found high levels of emotional exhaustion in 60%, high levels of depersonalization in 36% and low levels of personal accomplishment in 19% of pain physicians [31]. The study found similar rates of high emotional exhaustion and low personal accomplishment between anesthesia and physiatry, though physiatry reported higher incidences of depersonalization.

Our results are concerning both for their direct and indirect implications. First, the proportion of physicians who considered leaving or changing their career due to the collection and utilization of PSS – nearly a third of respondents – is concerningly high. In the setting of a worsening opioid epidemic, several society and state guidelines have recommended that physicians consider referral to a pain specialist when managing patients at elevated risk of overdose or other adverse event [32–34]. As this and related trends continue, pain medicine-trained physicians will prove central in reducing opioid overdose-related deaths. Introducing pressure toward career change via misuse of patient satisfaction data is a direct threat to that success.

Surveys, both as utilized in this study’s design and those utilized in measuring patient satisfaction, introduce the potential for responder bias. A limitation of this study is the binary (yes/no) response requirement of many of the survey’s questions; this approach was chosen to facilitate ease of response. The response rate of the survey was 34% among those who clicked on the link within the email, and only 20% of physicians who viewed the email clicked the link, which compares favorably to response rates for some patient satisfaction surveys [18] While only soliciting responses from members of a single professional medical society may limit the generalizability of these findings, the responding group represented multiple different specialties, a wide range of experience and even included a number of international responders. Most responders were male, which may reflect the makeup of the field at present or under-represent the views of our female colleagues. The field of pain medicine anesthesiology may have been relatively under-represented compared with PM&R.

## Conclusion

The collection of PSS is associated with decreased job satisfaction and increased burnout in physicians treating patients with spine-related pain. Given the increasing concern regarding the negative impacts of physician burnout, further research is warranted to determine the extent to PSS may be contributing to the trend.

## Future perspective

Given the increasing concern regarding the negative impacts of physician burnout, further research is warranted to determine the extent to PSS may be contributing to the trend. The findings in our study highlight that many physicians may feel discordance between doing what is right and doing what the patient wants in order to not negative effect the patient satisfaction survey evaluating their performance. This indeed may lead to physician burnout but of more concern may lead to inappropriate diagnostic evaluations and interventional treatments. This indeed is of concern in pain management practices and warrants further study.

**Summary points**

- Physicians report feeling pressure to emphasize patient satisfaction scores (PSS).
- Physicians report PSS have a negative effect on job satisfaction.
- Physicians report PSS contribute to physician burnout.
- PSS are associated with decreased job satisfaction and increased burnout.
